# The circRNA circSIAE Inhibits Replication of Coxsackie Virus B3 by Targeting miR-331-3p and Thousand and One Amino-Acid Kinase 2

**DOI:** 10.3389/fcimb.2021.779919

**Published:** 2022-01-24

**Authors:** Qingru Yang, Yuhan Li, Yan Wang, Xiaorong Qiao, Tingjun Liu, Hua Wang, Hongxing Shen

**Affiliations:** ^1^ Medical College, Jiangsu University, Zhenjiang, China; ^2^ Clinical Laboratory, Jiangyin Municipal Center for Disease Control and Prevention, Jiangyin, China

**Keywords:** Coxsackie virus B3, circular RNAs, circSIAE, miR-331-3p, thousand and one amino-acid kinase 2

## Abstract

Coxsackie virus B3 (CVB3), an enterovirus, is the main pathogen causing viral myocarditis, pericarditis, hepatitis and other inflammation-related diseases. Non-coding RNAs with a closed loop molecular structure, called circular RNAs (circRNAs), have been shown to be involved in multiple virus-related processes, but roles and mechanisms in CVB3 infection have not been systematically studied. In this study, when HeLa cells were infected with CVB3, the expression of hsa_circ_0000367 (circSIAE) was significantly decreased as demonstrated by real-time quantitative PCR assays. We found that circSIAE downregulated the expression of miR-331-3p through direct binding and inhibited the replication of CVB3 in HeLa and 293T cells. The analysis of signals downstream of miR-331-3p suggested that miR-331-3p promotes CVB3 replication, viral plaque formation and fluorescent virus cell production through interactions with the gene coding for thousand and one amino-acid kinase 2 (*TAOK2*). In conclusion, this study found that circSIAE can target *TAOK2* through sponge adsorption of miR-331-3p to inhibit the replication and proliferation of CVB3 virus, providing an early molecular target for the diagnosis of CVB3 infection.

## Introduction

Coxsackievirus B3 (CVB3) is a member of the Picornaviridae family in the genus *Enterovirus*. It causes viral myocarditis, pancreatitis, and hand, foot and mouth diseases (Li et al., 2015). CVB3 uses its encoded protease to take over the translation machinery of the host cell and to direct ribosomes to the viral mRNA (Qiu et al., 2017). Therefore, CVB3 infection causes abnormal expression of multiple proteins and non-coding RNAs (He et al., 2013; Ursu et al., 2019). Conversely, host factors also exert an influence on CVB3 replication (Zell et al., 1995).

Non-coding RNAs (ncRNAs), including long non-coding RNA (lncRNA), microRNA(miRNA) and circular RNA (circRNA), play vital roles in many diseases. In CVB3 infection, in particular the virus can induce abnormal expression of several lncRNAs (Tong et al., 2019). In addition, host lncRNA has also been shown to affect viral replication and infection; for example, the lncRNA hypoxia-inducible factor 1-α-antisense 1 has been associated with pro-apoptotic and pro-inflammatory effects in CVB3-induced myocarditis through its targeting of miR-138 (Cao et al., 2020). Similarly, lncRNA AK085865 was shown to promote CVB3 to induce macrophage M2 polarization in patients with viral meningitis by regulating the formation of a complex between interleukin enhancer binding factors 2 and 3 that mediates miR-192 biogenesis (Zhang et al., 2020).

Another class of small non-coding RNAs, miRNA, is widely expressed and regulates the replication and pathogenesis of RNA viruses (Trobaugh and Klimstra, 2017). Regulation of miRNA expression has been used to study the effects of CVB3 infection on cell differentiation and development, and such studies have impacted the treatment of CVB3 infections (Yao et al., 2019). According to previous reports, one type of miRNA, miR-30a, modulates type I interferon responses to facilitate CVB3 replication via targeting of tripartite motif protein 25 (Li et al., 2020b). Another miRNA, miR-126, promotes coxsackievirus replication by mediating cross-talk of ERK1/2 and Wnt/β-catenin signal pathways (Ye et al., 2013). On the other hand, miR-21 has been shown to serve a protective role against CVB3 infection through targeting the MAP2K3/p38 MAPK signaling pathway (He et al., 2019).

Recently, multiple circRNAs have been shown to have increasingly complex biological functions that overlap with pathogenic viral mechanisms (Li et al., 2017). For example, it has been reported that both host and virus-derived circRNAs interact with antiviral proteins bound to double-stranded RNA (dsRNA) and to modulate the immune response during host-virus interactions (Awan et al., 2021). Similarly, the circRNA circ_0000479 has been shown to indirectly regulate expression of retinoic acid-inducible gene I through sponging of miR-149-5p, thus blocking viral replication (Lu et al., 2020). In general, many circRNAs influence the pathogenesis of infectious diseases (Zhang et al., 2019b; Zhao et al., 2019) and confer protection against viral infection by regulating gene expression in host cells (Liu et al., 2019; Yan and Chen, 2020). However, the mechanisms of action of circRNAs in CVB3 infection and replication remain unclear.

CircSIAE (hsa_circ_0000367) is a circRNA formed by reverse splitting of linear SIAE gene at the position chr11:124517260-124518071 on chromosome, consisting of 244 nucleotides. It is derived from a spline of SIAE mRNA associated with immune diseases and reported as a novel potential biomarker for emerging rheumatoid arthritis (Luo et al., 2020; Zhou et al., 2020). In this study, we used cultured cell systems to study the effect of CVB3 infection on expression levels of circRNAs and to investigate potential downstream mechanisms of their involvement in viral processes. When HeLa cells were infected with CVB3, we found that the expression level of circSIAE was significantly decreased. Moreover, we investigated the effects of an axis involving circSIAE, miR-331-3p, and thousand and one amino-acid kinase 2 (TAOK2) on CVB3 replication, and we thus revealed a mechanism by which circSIAE regulates CVB3 replication and proliferation by acting as a competitive endogenous RNA (ceRNA).

## Materials and Methods

### Cell Culture and Transfection

Human cervical cancer cells (HeLa) and 293T cells (a gift from Dr. Huaiqi Jing, Chinese Center for Disease Control and Prevention, Beijing, China) were cultured in Dulbecco’s modified Eagle’s medium (Thermo Fisher Scientific Inc., Waltham, MA, USA) containing 10% fetal bovine serum (Thermo Fisher Scientific Inc., Waltham, MA, USA). All cells were incubated at 37 °C with 5% CO_2_. The Nancy strain of CVB3 was donated by Professor Chen Ruizhen, Zhongshan Hospital, Fudan University, China. A plasmid containing the CVB3 complete gene complementary sequence, capable of expressing enhanced green fluorescent protein (CVB3-eGFP) was a gift from Professor Zhaohua Zhong, Harbin Medical University, China. Circular RNA molecules were transfected with small interfering RNAs, including si-circSIAE-1, si-circSIAE-2 and negative control (si-NC), or overexpressed as plasmids, including pcicR-circSIAE and no-load pcicR-3.0, miR-331-3p mimics, miR-331-3p inhibitor, miRNA negative control (miR-NC and in-miR-NC) (Jima, Suzhou, China), si-TAOK2-2804, si-TAOK2-1065 or siRNA negative control (si-NC). Oligonucleotides (50 nM) were transfected into cells using Lipofectamine 3000 Reagent (Invitrogen, Carlsbad, CA, USA) according to the manufacturer’s instructions. Oligonucleotide sequences are provided in **Table S1**.

### Cytoplasmic Nucleus Separation

Nuclear plasma was extracted with cytoplasmic nucleus extraction kit (Thermo Fisher, USA) according to the manufacturer’s instructions. Briefly, HeLa cells were harvested and washed in PBS. Then, cells were suspended in 100–500 μL ice-cold Cell Fractionation Buffer and incubated on ice for 5–10 min. After centrifugation, the supernatant was the cytoplasmic fraction and the precipitation was the nuclear pellet. The nuclear pellet was suspended in Cell Disruption Buffer. 2× Lysis/Binding Solution was added to the cytoplasmic fraction and the nuclear pellet. Finally, the cytoplasmic and nuclear fractions were washed in Wash Solution, eluted with Elution Solution and stored at –80°C.

### Quantitative Real-Time PCR

The total RNA was extracted from HeLa cells using Trizol (Sigma-Aldrich, Saint Louis, MO, USA) and reversed transcribed into cDNA using an RNA reverse transcription kit (TaKaRa Bio Inc., Shiga, Japan). The transcripts were quantified according to the manufacturer’s instructions (TaKaRa Bio Inc., Shiga, Japan).

### Western Blot Analyses

Total protein was extracted from HeLa cells using RIPA lysis buffer (Thermo Fisher, USA). Proteins were separated using SDS-PAGE and then transferred to polyvinylidene fluoride membranes, which were blocked with 0.5% skimmed milk and then incubated with primary and secondary antibodies. The antibodies used in this study included antibodies against nuclear factor (NF)-κB (Cell Signaling Technology (CST), Danvers, MA, USA), phosphorylated NF-κB (p-NF-κB) (CST, Danvers, MA, USA), extracellular signal-regulated kinase (ERK)1/2 (CST, Danvers, MA, USA), p-ERK1/2 (CST, Danvers, MA, USA), and anti-GAPDH (Abcam, Cambridge, UK). The secondary antibodies were anti-rabbit IgG (Jackson ImmunoResearch, Inc., PA, USA) antibodies. VP-1, used at a dilution of 1:2000, is a goat polyclonal secondary antibody to mouse IgG (Sino Biological). Protein signals were detected using a BeyoECL detection instrument and an ECL chemiluminescence kit (BeyoTime, China), and protein bands were ultimately scanned with ImageJ software in grayscale mode.

### Dual-Luciferase Reporter Assay

The circSIAE and miR-331-3p fragments containing the target binding site (wild-type, WT) or mutated binding site (mutated, MUT) were cloned into the pmirGLO vector (Suzhou Jimar, China). These plasmids as well as miR-331-3p mimics and inhibitors were transfected into 293T cells using Lipofectamine 3000 (Invitrogen, Shanghai, China). After 48 h, dual luciferase activity in 293T cells was examined with a dual luciferase reporter assay system (Vazyme, Nanjing, China).

### Virus Plaques

Virus plaques assay was performed as described previously (Liu et al., 2021). Briefly, HeLa cells were inoculated in 12 well plates at a density 1.5 × 10^5^/well. After culturing overnight, virus-containing samples serially diluent was added. The cells were incubated at 37°C for 1 h and the virus solution was aspirated. Then the cells were overlaid with low melting point agarose (0.75%), cultured at 37°C for 72 h, and were fixed with fixative for 30 min. Finally, cells were stained with crystal violet (1%) for 30min and washed with PBS for three times. The stained cells were observed and recorded with visible microscopy.

### Statistical Analysis

In each experiment, all determinations were performed at least in triplicate. Statistical analysis was performed using the GraphPad Prism 5.0 (GraphPad Software Inc., San Diego, CA, USA). All data are expressed as the mean ± standard deviation (X ± SD), and the significance of difference was detected using t test or one-way analysis of variance (ANOVA), with p<0.05 considered significant difference.

### Bioinformatics Analysis

Three databases, CircBank (http://www.circbank.cn/), the Cancer Specific CircRNA Database (CSCD, http://gb.whu.edu.cn/CSCD/) and Circinteractome (https://circinteractome.nia.nih.gov/), were used to investigate downstream target miRNAs of circSIAE. The databases miRPathDB (https://mpd.bioinf.uni-sb.de/), TargetScan (http://www.targetscan.org/vert_72/) and Starbase (https://starbase.sysu.edu.cn/agoClipRNA.php?source=mRNA) were used to predict downstream target genes of miR-331-3p. Gene Ontology (GO) analysis was carried out to investigate biological process, molecular function, and cellular component.

## Results

### The circRNA circSIAE Exhibits a Cyclic Structure and Inhibits CVB3 Replication

**Figure 1:**
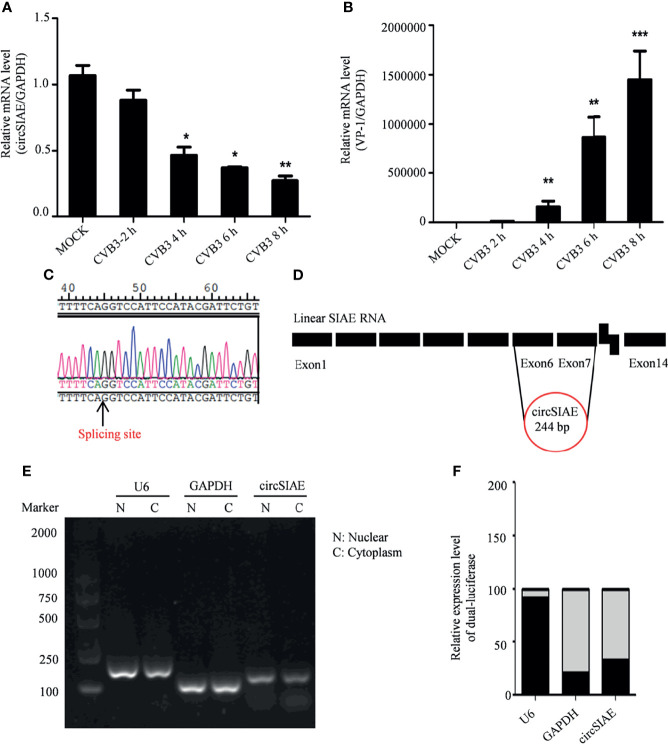
Genomic structure and location of circSIAE. **(A, B)** Total RNA was extracted at 2, 4, 6 and 8 h post-infection with CVB3, and the expression levels of circSIAE and VP-1 at mRNA level were analyzed by qRT-PCR. **(C)**. The splice sites of circSIAE were detected by sequencing. **(D)**. The circSIAE genome is formed by reverse splicing of exons 6 and 7 of linear SIAE. **(E, F)** Total RNA was isolated from HeLa cell cytoplasm and nucleus, *via* a nucleoplasmic separation kit. GAPDH and U6 were used as cytoplasmic and nuclear markers. The expression level of circSIAE in the cytoplasm was verified by agarose electrophoresis **(E)** and qRT-PCR **(F)**. *p < 0.05, **p < 0.01, ***p < 0.001.

Total RNA of HeLa cells infected with CVB3 at a multiplicity of infection of 5 was extracted at 2 h, 4 h, 6 h and 8 h post-infection to identify differential expression of circSIAE. Uninfected HeLa cells were used as a control. Quantification of RNA expression by qRT-PCR showed that circSIAE expression decreased with CVB3 infection time, and the effect was most obvious at 6 h (**Figures 1A, B**).

To investigate the structure of the circSIAE molecule, we explored the cleavage sites leading to the formation of this circRNA with the sequencing technology (**Figure 1C**). As shown in **Figure 1D**, analysis of the cleavage sites is consistent with the formation of the mature circSIAE molecule by reverse splicing of exons 6 and 7 of linear SIAE (**Figure 1D**).

The function of the circSIAE molecule depends in part on the subcellular localization of the molecule. Therefore, the cytoplasm and nucleus were separated with a nucleoplasmic separation kit, and agarose gel electrophoresis and qRT-PCR were used to analyze the distribution of the molecule in the nucleus and cytoplasm, with the U6 small nuclear RNA serving as a marker of the nucleus and the GAPDH serving as a marker of the cytoplasm. Here, circSIAE was found to be mainly distributed in cytoplasm, accounting for 79% of the total circSIAE (**Figures 1E, F**).

**Figure 2:**
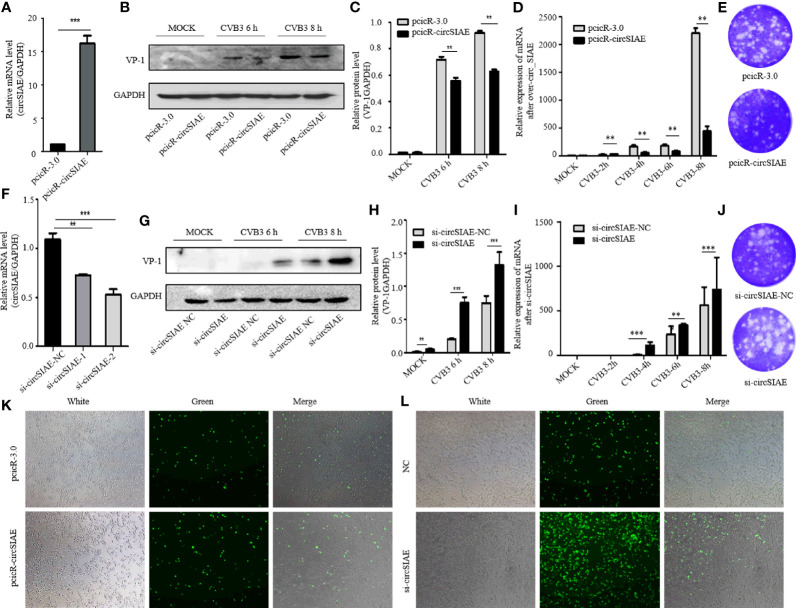
The effect of circSIAE on CVB3 replication. **(A)** After transfection of no-load pcicR-3.0 and overexpression of pcicR-circSIAE in HeLa, total RNA was extracted 36 h post-transfection, and the success of overexpression of circSIAE was verified by qRT-PCR. **(B)** After 36 hours post-transfection with pcicR-3.0 and pcicR-circSIAE, HeLa cells were infected with CVB3 (multiplicity of infection = 5), and total protein was extracted at 6 and 8 h post-infection. The expression of VP-1was detected by Western blot. **(C, H)** Protein levels of VP-1 were quantified by densitometric analysis using ImageJ, normalized to GAPDH and presented as fold changes compared to the control group. **(D)** After 36 hours post-transfection with pcicR-3.0 and pcicR-circSIAE, HeLa cells were infected with CVB3 and total RNA was extracted at 2, 4, 6 and 8 h post-infection. The expression of *VP-1* was detected by qRT-PCR. **(E, J)** To verify the effect of circSIAE on CVB3 proliferation, cells were infected with CVB3 36 hours post-transfection with no-load pcicR-3.0 and circSIAE (NC, si-circSIAE-2), and viral supernatant was collected at 6 hours post-infection for viral plaque tests. **(F)** The knockdown efficiency of circSIAE was verified by qRT-PCR. **(G)** Cells were transfected with empty vector (NC) and si-circSIAE-2 and then infected withCVB3. Total protein was isolated at 6 and 8 h post-infection. Western blotting was used to analyze virus replication. **(I)** Cells were transfected with empty vector (NC) and si-circSIAE-2 and then infected withCVB3. Total RNA was isolated at 2, 4, 6 and 8 h post-infection. The expression of *VP-1* was detected by qRT-PCR. **(K, L)** After 293T cells were co-transfected with CVB3-eGFP, pcicR-3.0/pcicR-circSIAE and NC/si-circSIAE-2, the fluorescence of infected cells was observed by fluorescence microscopy, and the effect of circSIAE on CVB3 replication was analyzed. **p < 0.01, ***p < 0.001.

In order to investigate possible impacts of the decreased expression of circSIAE, several constructs were transfected into HeLa cells to induce overexpression or silencing of the circRNA. First, to confirm ectopic expression, total RNA was extracted from HeLa cells that were transfected with pcicR-circSIAE overexpression vector, with empty pcicR-3.0 as a control, at 48 h post-transfection, and circSIAE overexpression was verified by qRT-PCR (**Figure 2A**). Moreover, circSIAE expression was suppressed with small interfering RNAs, si-circSIAE-1 and si-circSIAE-2. When these constructs were transfected into HeLa cells and qRT-PCR analysis of total RNA was performed, it was found that si-circSIAE-2 exhibited a relatively high knockdown efficiency as compared to the NC (**Figure 2F**). Furthermore, the si-circSIAE-2 and the pcicR-circSIAE were only influenced by the circSIAE but not by the linear SIAE (**Supplementary Figure 2**). Then, when circSIAE was overexpressed or silenced, HeLa cells were infected with CVB3, and the virus supernatant was collected and total cell protein was extracted at 6 and 8 h post-infection.

Subsequent, Western blotting analyses demonstrated that overexpression of circSIAE inhibited the expression of a major viral capsid protein, VP-1 (**Figures 2B, C**), while knockdown of circSIAE promoted the expression of VP-1 (**Figures 2G, H**). To confirm the function of circSIAE on CVB3 proliferation, viral plaque analysis was performed. The results showed that, as compared with the control group, overexpression of circSIAE decreased the number of viral plaques, while the number of viral plaques from supernatants of HeLa cells with knocked down circSIAE increased, suggesting that circSIAE can inhibit the proliferation and/or release of CVB3 (**Figures 2E, J**).

Furthermore, to confirm the function of circSIAE on CVB3 proliferation curve, the expression of *VP-*1 by qRT-PCR was performed. CircSIAE was overexpressed or silenced, HeLa cells were infected with CVB3 at 2, 4, 6 and 8 h. As shown in **Figure 2D**, the expression of *VP-*1 was decreased in the overexpression cells in contrast to the control cells. Meanwhile, circSIAE knockdown significantly increased the expression of *VP-*1(**Figure 2I**).

In order to directly observe the influence of circSIAE on CVB3 replication in a time-dependent manner, CVB3 that expressed eGFP was infected into 293T cells at a multiplicity of infection of 0.1, and the impacts of circSIAE overexpression and knockdown were determined. After 48 to 72 h post-infection, the number of fluorescent cells was observed by fluorescence microscopy. The fluorescence intensity of circSIAE-overexpressing cells was lower than that of the control group, while the fluorescence intensity of the circSIAE knockdown group was higher than that of the control group (**Figures 2K, L**).

Taken together, then, the results of VP-1 expression, viral particle production, and CVB3-eGFP infection demonstrated that circSIAE inhibits the replication and proliferation of CVB3.

### miR-331-3p Is a Sponge for circSIAE and Promotes CVB3 Replication

**Figure 3:**
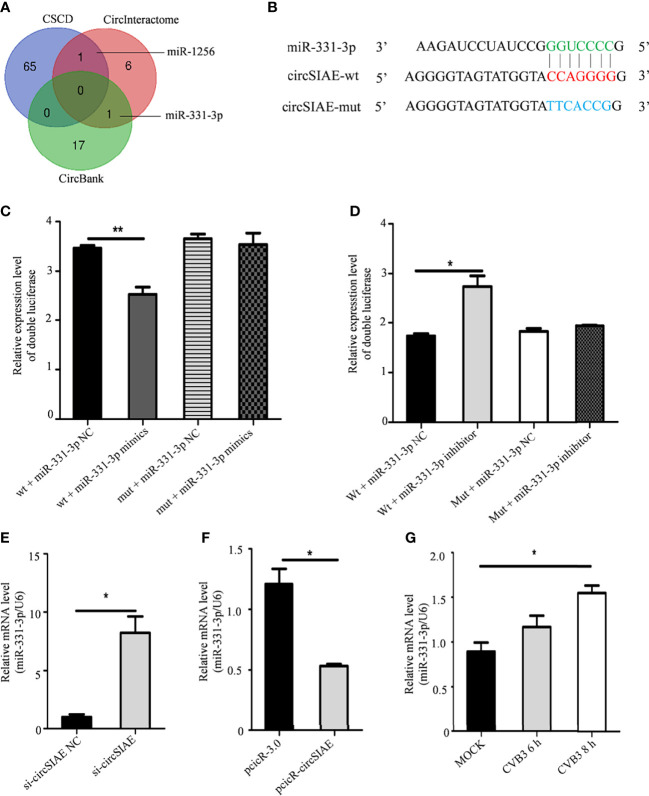
The search for downstream target miRNAs of circSIAE. **(A)** Potential downstream target miRNAs of circSIAE were searched for in bioinformatics databases. **(B)** Wild-type and mutant sequences of circSIAE. **(C, D)** After 293T cells were transfected with NC/mimics/inhibitor of miR-331-3p and wild type, and circSIAE was knocked down for 24 h, the ratio of firefly luciferase to *Renilla* luciferase was measured with a dual luciferase kit. **(E, F)** Total RNA was extracted from HeLa cells after transfection with pcicR-circSIAE/si-circSIAE for 36 h, and the expression level of miR-331-3p was detected by qRT-PCR. **(G)** qRT-PCR was used to verify the relationship between miR-331-3p and CVB3. *p < 0.05, **p < 0.01.

Using CircBank, CSCD and Circinteractome databases, miR-331-3p was found to be a potential downstream target of circSIAE (**Figure 3A**). A double luciferase assay was used to test for the existence of binding sites that mediate an interaction between miR-331-3p and circSIAE (**Figure 3B**). The results showed that the expression of wild-type circSIAE was decreased when a miR-331-3p mimic was expressed. Similarly, the expression of wild-type circSIAE increased when an miR-331-3p inhibitor was present (**Figures 3C, D**).

To further verify the downstream target miRNA of circSIAE, total RNA was extracted after overexpression or knockdown of circSIAE. Quantification of miR-331-3p expression by qRT-PCR showed that overexpression of circSIAE resulted in reduced expression of miR-331-3p, while knockdown of circSIAE increased miR-331-3p expression (**Figures 3E, F**). In addition, total RNA was extracted 6 to 8h after CVB3 infection, and qRT-PCR demonstrated that miR-331-3p increased with CVB3 infection time (**Figure 3G**). Because CVB3 infection correlates with decreased circSIAE expression (**Figure 1**), increases in miR-331-3p with CVB3 expression are consistent with a targeting of miR-331-3p by circSIAE.

**Figure 4:**
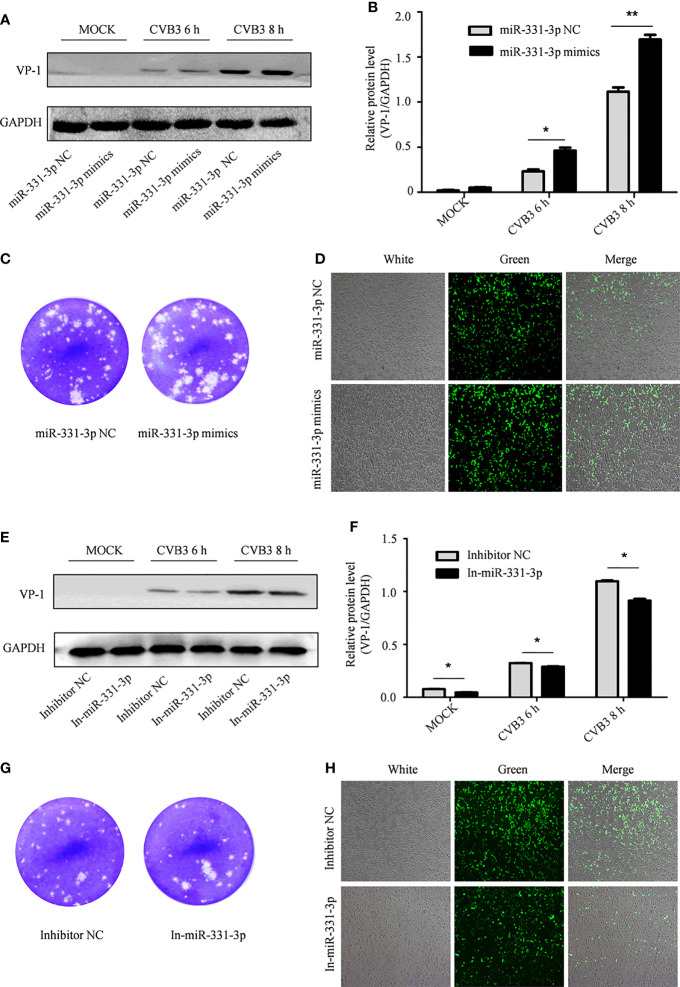
The effect of miR-331-3p on the replication and proliferation of CVB3. **(A, E)** After transfection with miR-331-3p mimics/inhibitor for 36 h, cells were infected with CVB3 for 6 or 8 h, and total protein was extracted, and the effect of miR-331-3p on CVB3 replication was investigated by Western blot. **(B, C, F, G)** HeLa cells were infected with CVB3 36 h after transfection with miR-331-3p mimics/inhibitor. The viral supernatant after 6 h of infection was collected and used to infect HeLa cells for 1 h. The cells were solidified with low melting point agarose for 2 hours and fixed with 75% glacial acetic acid for 48 hours. **(D, H)** 293T cells co-transfected with CVB3-eGFP and miR-331-3p mimics/inhibitor were observed by fluorescence microscopy. *p < 0.05, **p < 0.01.

In order to further test the effect of miR-331-3p on CVB3, miR-331-3p mimics and inhibitors were transfected into HeLa cells. Uninfected HeLa cells were used as controls. Western blot analyses and viral plaque assays showed that miR-331-3p mimics promoted the expression of VP-1 and correlated with increased numbers of viral plaques compared with the control group. Expression of an miR-331-3p inhibitor inhibited VP-1 expression and decreased viral plaque production (**Figures 4A–C, E–G**).

Similarly, 293T cells were co-transfected with CVB3-eGFP and miR-331-3p mimics or inhibitors, and the numbers of fluorescent cells were measured by fluorescence microscopy. Expression of miR-331-3p mimics increased the number of fluorescent cells (**Figure 4D**), while expression of miR-331-3p inhibitors was found to decrease the number of fluorescent cells (**Figure 4H**). In summary, analyses of VP-1 protein expression and production of viral particles in HeLa cells and viral activity in 293T cells confirmed that miR-331-3p promotes the replication and proliferation of CVB3, and a double luciferase assay confirmed that miR-331-3p is a downstream target miRNA of circSIAE.

### *TAOK2* Is a Downstream Target Gene of miR-331-3p and Suppresses CVB3 Replication

**Figure 5:**
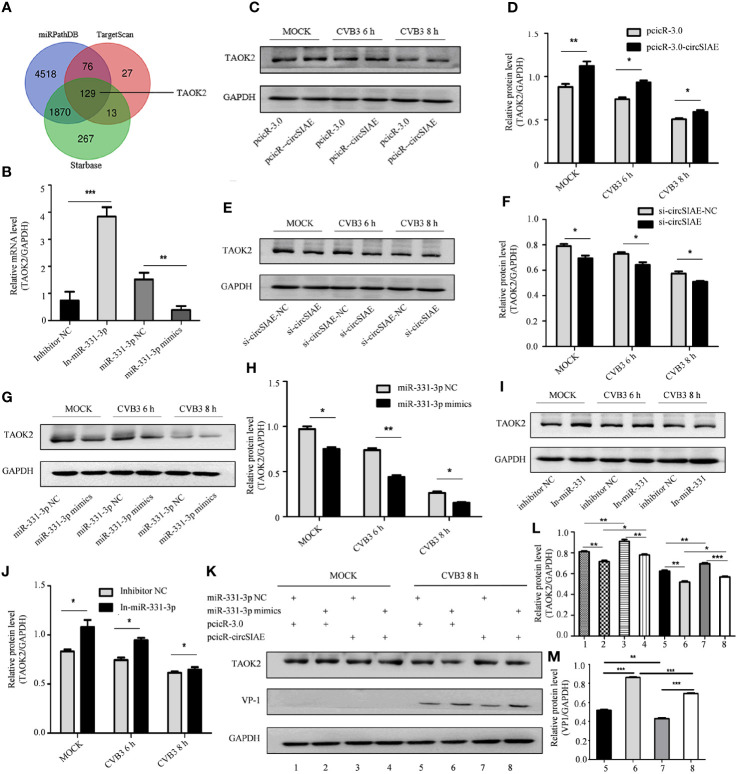
Searching for downstream target genes of miR-331-3p. **(A)** Downstream target genes of miR-331-3p were identified by database screening. **(B)** Total RNA was extracted after transfection with miR-331-3p mimics/inhibitor for 36 h, and the expression level of *TAOK2* was verified by qRT-PCR. **(C, E, G, I)** After circSIAE and miR331-3p were overexpressed/knocked down in HeLa cells, the relationship between circSIAE/miR-331-3p and TAOK2 was verified by Western blotting and qRT-PCR analysis. **(D, F, H, J)** The protein levels were quantified by densitometric analysis using ImageJ. **(G, K)** Total protein of HeLa cells was extracted after co-transfection of pcicR-3.0-circSIAE and miR-331-3p mimics, and cells were then infected with CVB3 for 8 h, and protein changes of TAOK2 and VP-1 were determined by Western blot. **(L, M)** The protein levels of figures of **(J, K)** were quantified by densitometric analysis using Image J. *p < 0.05, **p < 0.01, ***p < 0.001.

Four databases, including miRDB, miRTarBase, miRPathDB and TargetScan, were used to predict downstream target genes of miR-331-3p, and the gene encoding TAOK2 was identified as a prominent hit (**Figure 5A**). The relationship between *TAOK2* and miR-331-3p that was predicted *via* bioinformatics analyses was tested by analysis of expression of *TAOK2* mRNA by qRT-PCR. Here, the results showed that, as compared with the control group, the expression of *TAOK2* mRNA was decreased upon transfection of miR-331-3p mimics, while the expression of *TAOK2* mRNA was increased upon transfection of miR-331-3p inhibitors (**Figure 5B**).

The relationship was also tested in the context of CVB3 infection. After transfection of individual constructs directing the overexpression or knockdown of circSIAE or with constructs expressing miR-331-3p mimics or inhibitors in HeLa cells, the cells were infected with CVB3. Proteins were extracted after CVB3 infection at 6 h and 8h, and the relationship between TAOK2 protein expression and the status of circSIAE and miR-331-3p was determined by Western blotting. As shown in **Figures 5C–J**, the transfection of an miR-331-3p mimic or knockdown of circSIAE inhibited the expression of TAOK2, while transfection of an miR-331-3p inhibitor or overexpression of circSIAE promoted the expression of TAOK2.

These relationships were also tested by co-transfection of the circSIAE-related constructs with those affecting miR-331-3p activity in a similar manner. Western blotting demonstrated the combined effects of circSIAE and miR-331-3p on TAOK2, as shown in **Figure 5K**. These results confirmed that expression of the *TAOK2* gene was positively correlated with expression of circSIAE and negatively correlated with activity of miR-331-3p (**Figure 5K**).

**Figure 6:**
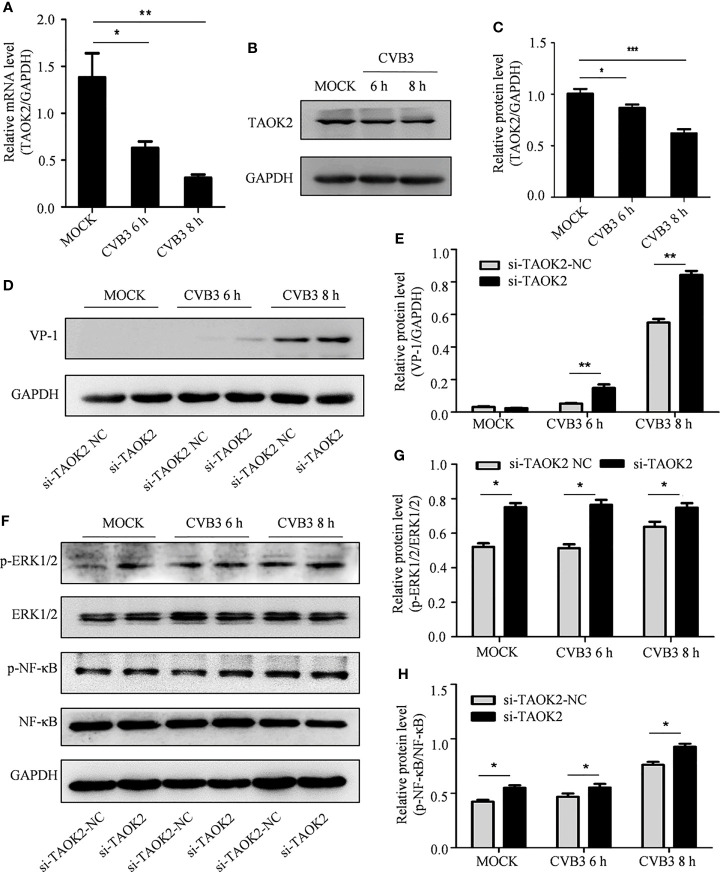
The effect of TAOK2 on CVB3 replication. **(A)** Total RNA was extracted from HeLa cells infected with CVB3 for 6 and 8 h, and the relationship between TAOK2 and CVB3 was investigated by qRT-PCR. **(B)** Total protein was extracted from HeLa cells 6 and 8 hours post-infection with CVB3. Western blotting was used to verify the relationship between TAOK2 and CVB3. **(C, E, G, H)** Same as **Figures 2C, H**. **(D)** Total protein of si-TAOK2-transfected cells infected with CVB3 at 6 and 8 h was extracted, and the relationship between TAOK2 and VP-1 was verified by Western blotting. **(F)** Total protein was extracted from si-TAOK2 transfected HeLa cells following for 6 and 8 h of infection with CVB3. The levels of p-ERK1/2, ERK1/2, p-NF-κB and NF-κB were detected by Western blot. *p < 0.05, **p < 0.01, ***p < 0.001.

As CVB3 infection correlates with decreased expression of cellular circSIAE, it was predicted that CVB3 infection would also correlate with decreased expression of the gene encoding TAOK2. HeLa cells were infected with CVB3, and the proteins and total RNA of infected and uninfected cells were collected at 6 and 8 h post-infection. Western blot (**Figure 6A**) and qRT-PCR (**Figures 6B, C**) were performed to test the impact of CVB3 on *TAOK2* protein and mRNA. As predicted, CVB3 infection correlated with reduced expression in a time-dependent manner (**Figures 6A–C**).

The impact of expression of TAOK2 protein on CVB3 activity was further investigated by knocking down expression of *TAOK2* with siRNA technology. Two siRNA constructs, si-TAOK2-2804 and si-TAOK2-1065, were created and transfected into HeLa cells. Subsequent Western blotting analysis demonstrated that si-TAOK2-2804 was effective at knocking down expression of *TAOK2* (**Supplementary Figure 1**). Therefore, si-TAOK2-2804 was transfected into HeLa cells prior to infection with CVB3, and total protein was extracted 6 and 8 h after infection. Western blot results showed that VP-1 expression increased after *TAOK2* was knocked down, suggesting that TAOK2 inhibited CVB3 replication (**Figures 6D, E**). To verify the relationship of TAOK2 with several virus-associated signaling pathways, the levels of p-ERK1/2, ERK1/2, p-NF-κB and NF-κB were detected by Western blot. As shown in **Figure 6F–H**, transfection with si-TAOK2 promoted the phosphorylation of ERK1/2 and NF-κB, consistent with a model in which TAOK2 downregulates signaling through ERK1/2 and NF-κB.

### Inhibition of CVB3-Induced Inflammation by the circSIAE/miR-331-3p/TAOK2 Axis

**Figure 7:**
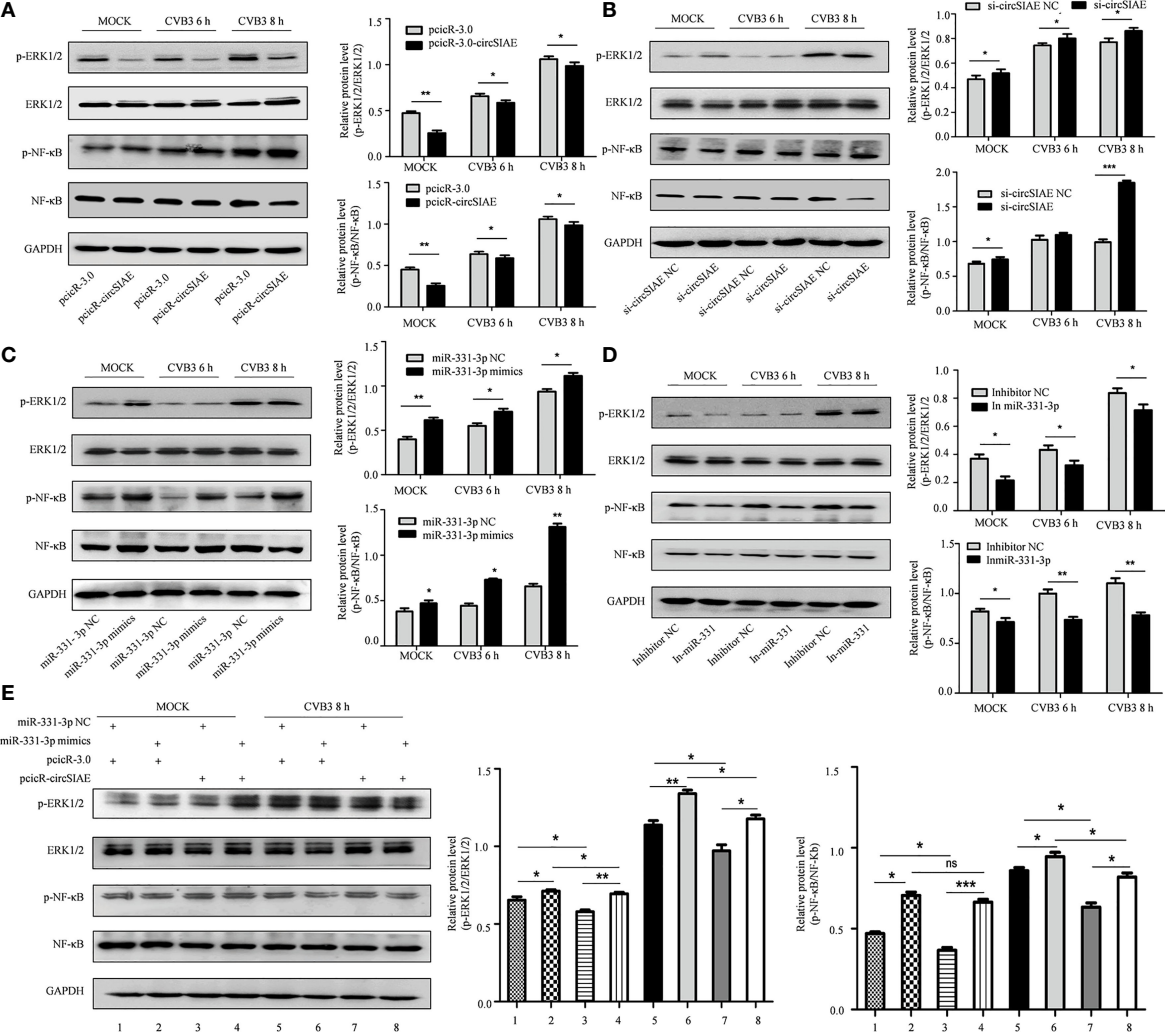
The effect of the circSIAE/miR331-3p/TAOK2 axis on the ERK/NF-κB inflammation pathway. **(A–D)** After transfection with pcicR-circSIAE/si-circSIAE/miR-331-3p mimics/miR-331-3p inhibitor and its control for 36 h, total protein of cells infected with CVB3 for 6 h and 8 h were extracted. The levels of p-ERK1/2, ERK1/2, p-NF -κB and NF-κB were detected by Western blot. The histograms on the right showed the levels of protein as quantified by densitometric analysis using ImageJ. **(E)** Total protein of cells transfected with pcicR-3.0-circSIAE and miR-331-3p were extracted following 8 h of infection with CVB3. The relationship between ERK1/2 and NF-κB protein was verified by Western blot. *p < 0.05, **p < 0.01, ***p < 0.001, ns, no statistically significance..

To test the impact of these molecules on CVB3 activity, transcripts directing the overexpression or knockdown of circSIAE and miR-331-3p mimics or inhibitors were transfected individually into HeLa cells for 36 h, and the cells were then infected with CVB3. At 6 and 8 h post-infection, total cell proteins were extracted. Western blot analysis showed that knockdown of circSIAE and expression of an miR-331-3p mimic promoted the replication of CVB3; in addition, levels of p-NF-κB and p-ERK1/2 tended to increase compared with the control group (**Figures 7B, C**). Conversely, compared with the control group, circSIAE overexpression and expression of an miR-331-3p inhibitor inhibited CVB3 replication and correlated with decreased p-NF-κB and p-ERK1/2 (**Figures 7A, D**).

Co-transfection of the circSIAE and miR-331-3p constructs was also performed. The resulting expression analyses showed that phosphorylation of NF-κB and ERK1/2 increased in cells transfected with miR-331-3p mimics group relative to control. The levels of p-NF-κB and p-ERK1/2 were also higher upon overexpression of circSIAE with miR-331-3p mimics. The levels of p-NF-κB and p-ERK1/2 were decreased upon overexpression of circSIAE alone. The levels of p-NF-κB and p-ERK1/2 decreased upon overexpression of circSIAE with miR-331-3p mimics relative to cells in which miR-331-3p mimics were expressed without overexpression of circSIAE (**Figure 7E**).

Together, these results indicate that overexpression of circSIAE inhibits the expression of *TAOK2* by targeting miR-33-3p and that this activity inhibits pathways leading to inflammation, while knockdown of circSIAE promotes the expression of *TAOK2* by targeting miR-33-3p and thus activates pathways leading to inflammation. Thus, circSIAE inhibits the replication and proliferation of CVB3 and influences an inflammatory response pathway by targeting miR-331-3p/TAOK2.

## Discussion

Several roles played by circRNAs in human cancers have been elucidated. These roles include acting as miRNA sponges (Zhang et al., 2019a; Han et al., 2020; Li S. et al., 2020) and encoding proteins (Zheng et al., 2019; Li et al., 2020a; Pan et al., 2020), and these functions tend to promote tumor progression. As these functions have become more established, our understanding of mechanisms of cancer progression have evolved, as well. However, studies of the functions of circRNAs in host-virus interactions are still in their preliminary stages.

The involvement of circRNAs in various viral infections has been established. Recently, for example, it was reported that after infection with hepatitis C virus, circEXOSC and circTIAL are up-regulated and promote viral infection. Another circRNA, circPSD3, has been shown to affecte RNA amplification at a post-translational step (Chen et al., 2020). In addition, multiple circRNAs were found to be differentially expressed in mice with acute respiratory distress syndrome induced by infection with influenza A virus as compared to control (Zhu et al., 2020). In particular, the expression of circRNA *Slco3a1* was significantly increased and circRNA *Wdr33* was markedly reduced in patients with acute respiratory distress syndrome (Wang et al., 2021). Zhu et al. showed that in hepatitis B virus-related hepatocellular carcinoma (HCC), the level of plasma circ_0027089 expression was decreased in comparison with cirrhosis patients and healthy participants (Zhu et al., 2020). Despite the connection of circRNA expression to viral infection, few studies focus on the function of circRNAs in virus replication. Here, our research elucidated the relationship between circSIAE and the replication of CVB3, and further identified circSIAE to play an important role as a miRNA sponge.

In this study, we found that the expression of circSIAE was decreased in HeLa cells infected with CVB3 and could inhibit the replication and proliferation of CVB3 in human cells. These functions were demonstrated by both virus plaque assays in HeLa cells and a CVB3-eGFP infection assay in 293T cells. Because previous research has demonstrated circRNA-miRNA-mRNA networks linking Hantaan virus and host cells as well as a sponging effect of circ_000479 on miR-149-5p in the regulation of viral replication (Lu et al., 2020), we predicted that the mechanism by which circRNA inhibited the replication of CVB3 could involve action as an miRNA sponge. According to CircBank, CSCD and Circinteractome databases, miR-331-3p was identified as a downstream target of circSIAE, and a dual luciferase assay supported the existence of sites of binding between miR-331-3p and circSIAE.

Interestingly, miR-331-3p has been shown to regulate the expression of amyloid β peptide (Aβ)_1–40_ (Liu and Lei, 2021) and inflammatory responses by regulating the expression of NLR family pyrin domain containing 6 (Nie et al., 2020). Importantly, miR-331-3p can inhibit replication of porcine reproductive and respiratory syndrome virus *via* targeting of ORF1b (You et al., 2020). Therefore, the effect of miR-331-3p on CVB3 replication was examined further.

It was found through virus plaque assays, Western blotting and a CVB3-eGFP infection assay, that down-regulation of miR-331-3p was able to decrease infectious viral particle formation, VP-1 protein synthesis, and the number of infected cells in comparison with the control. In contrast, these quantities were increased upon overexpression of miR-331-3p in cells, as compared with the control. Further functional studies showed that *TAOK2* was the downstream target gene of miR-331-3p. *TAOK2* is one of the genes at locus 16p11.2, and it encodes a serine/threonine kinase. Expression of *TAOK2* not only promotes the differentiation of mitotic cells, spindle localization and long-term disruption of intercortical neurons (Garg et al., 2020), but it also participates in infections by *Listeria monocytogenes* and several viruses (Quereda et al., 2020).

In order to further verify the involvement of the axis consisting of circSIAE/miR-331-3p/TAOK2, the production of TAOK2 protein was analyzed by Western blot in HeLa cells transfected with an miR-331-3p inhibitor or miR-331-3p mimics and by overexpressing or knocking down circSIAE. The results showed that transfection of miR-331-3p mimics and knockdown of circSIAE could inhibit the expression of TAOK2 protein, while transfection of miR-331-3p inhibitor and overexpression of circSIAE could promote the expression of TAOK2. Then, VP-1 and TAOK2 were measured by Western blotting in HeLa cells co-transfected with pcicR-circSIAE and miR-331-3p mimics, and the results showed that circSIAE and miR-331-3p were negatively correlated with TAOK2 expression and had a positive correlation with VP-1. In general, TAOK2 was negatively correlated with the expression of miR-331-3p and positively correlated with circSIAE. On the contrary, the expression of TAOK2 was decreased after transfection with miR-331-3p mimics, and it was found that circSIAE could positively regulate the expression of TAOK2, which further supported the hypothesis that circSIAE inhibited the replication of CVB3 through miR-331-3p/TAOK2. Finally, the results of investigations into inflammatory pathways showed that circSIAE inhibited the phosphorylation of NF-κB and ERK1/2 proteins.

In summary, our research shows that circSIAE can act as a sponge for miR-331-3p, inhibiting the progress of CVB3 virus replication by up-regulating *TAOK2*. Our research suggests a new mechanism for circSIAE to mediate the progress of CVB3 replication, and provides a theoretical basis for the study of circSIAE in CVB3 replication.

## Data Availability Statement

The original contributions presented in the study are included in the article/**Supplementary Material**. Further inquiries can be directed to the corresponding authors.

## Author Contributions

HW and HS designed the experiments. QY and YL performed the experiments. XQ, YW, and TL analyzed the data. QY wrote the paper. HW and HS reviewed and edited the manuscript. All authors read and approved of the manuscript.

## Funding

This work was supported by National Natural Science Foundation of China (grant 81971945).

## Conflict of Interest

The authors declare that the research was conducted in the absence of any commercial or financial relationships that could be construed as a potential conflict of interest.

## Publisher’s Note

All claims expressed in this article are solely those of the authors and do not necessarily represent those of their affiliated organizations, or those of the publisher, the editors and the reviewers. Any product that may be evaluated in this article, or claim that may be made by its manufacturer, is not guaranteed or endorsed by the publisher.
