# Supplementary material for: The circRNA circSIAE Inhibits Replication of Coxsackie Virus B3 by Targeting miR-331-3p and Thousand and One Amino-Acid Kinase 2
Source: Front Cell Infect Microbiol. 2022 Jan 24;11:779919. doi: 10.3389/fcimb.2021.779919 (PMC8820919; doi:10.3389/fcimb.2021.779919)
Supplement: Supplementary file 1 [file DataSheet_1.docx]

Table S1 Sequence details

| Primer | Sequence 5’-3’ |
| --- | --- |
| circSIAE-F- linear | CCTGCAGTACCGCAAGATGA |
| circSIAE-R- linear | CCTCCTCTTCCTCCTCTGGG |
| circSIAE-F- circ | GACTTGTCCAGGTCCATTCCA |
| circSIAE-R- circ | ACTCCCCCTGGTACCATACT |
| U6-F | CTCGCTTCGGCAGCACA |
| U6-R | AACGCTTCACGAATTTGCGT |
| 18S-F | CTTTCGATGGTAGGATAGTGGCCT |
| 18S-R | CAATGATCCTTCCGCAGGTTCACCTAC |
| GAPDH-F | ACCATCTTCCAGGAGCGAGAT |
| GAPDH-R | ATGACGAACATGGGGGCATC |
| VP-1-F | ATTCAAGGTCCGAGTCAAC |
| VP-1-R | CTGCTTGTCGTGTTA |
| si- circSIAE -F | CUUGUCCAGGUCCAUUCCATT |
| si- circSIAE -R | UGGAAUGGACCUGGACAAGTT |
| si-TAOK2-1065-F  si-TAOK2-1065-R  si-TAOK2-2804-F  si-TAOK2-2804-R | CCGGCAUCCCAACACCAUUTT  AAUGGUGUUGGGAUGCCGGTT  GGGAGGACCUGAACAAGAATT  UUCUUGUUCAGGUCCUCCCTT |

**Supplement Figure 1**

Total RNA and protein of si-TAOK2 transfected cells was extracted. qRT-PCR and Western blot were used to verify the inhibitory effect of TAOK2. It was found that si-TAOK2-2804 exhibited a relatively high knockdown efficiency as compared to the NC.


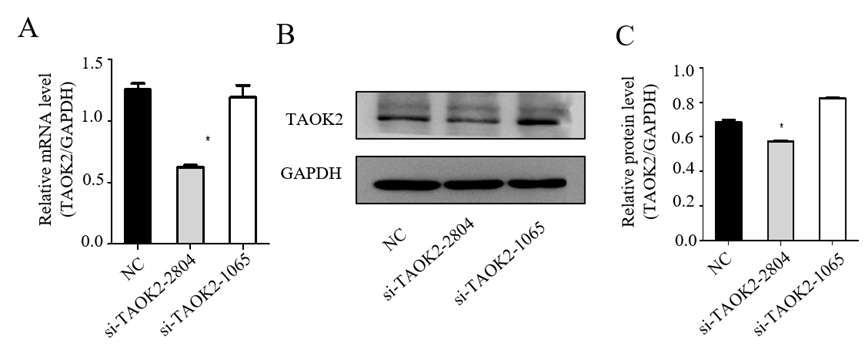


Fig 1. The inhibitory effect of si-TAOK2 was verified by qRT-PCR and Western blot

A. qRT-PCR was used to verify which TAOK2 could achieve TAOK2 knockout. B. The inhibitory effect of si-TAOK2 was verified by Western blot. C Same as figure 2C&G.

**Supplement Figure 2**

Total RNA was extracted from HeLa cells after transfection with pcicR-circSIAE/si-circSIAE. The expression level of circSIAE and the linear SIAE was detected by qRT-PCR. *p < 0.1, **p < 0.05, ***p<0.01. The results showed that the si-circSIAE and the plasmid over-expression were only influenced by the circSIAE expression but not by the linear SIAE.


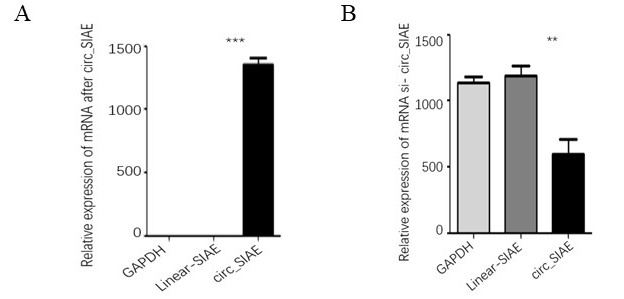


Figure 2 qRT-PCR was used to analyze the expressions of circSIAE and the linear SIAE.

A. B. The expression of circSIAE and the linear SIAE were detected by qRT-PCR after circSIAE was overexpressed or silenced.
